# Editorial: Recent advances in gaseous hydrocarbon sensing

**DOI:** 10.3389/fchem.2023.1249888

**Published:** 2023-07-17

**Authors:** Marilena Giglio, Vincenzo Spagnolo, Giansergio Menduni, Lei Dong, Weidong Chen

**Affiliations:** ^1^ PolySense Lab, Dipartimento Interateneo di Fisica, University and Politecnico of Bari, CNR-IFN, Bari, Italy; ^2^ State Key Laboratory of Quantum Optics and Quantum Optics Devices, Institute of Laser Spectroscopy, Shanxi University, Taiyuan, China; ^3^ Laboratoire de Physicochimie de l'Atmosphère, Université du Littoral Côté d'Opale, Dunkerque, France

**Keywords:** hydrocarbons detection, methane detection, gas sensing, photoacoustic spectroscopy, gas chromatography, on-chip silicon-on-insulator waveguide

Hydrocarbons occur in the environment in the form of natural gas, crude oil, and biomass or are produced through thermal processes. Because the combustion of these chemical compounds and their derivatives produces carbon dioxide, water, and heat, hydrocarbons are often employed as fuels. Hydrocarbon and methane isotopologue detection in the gas phase represents a powerful tool to guide oil exploration and production operations for the oil and gas industry (Guo et al., 2023; Olivieri et al., 2023). However, the transportation, storage, and refining processes of oil and natural gas have significant environmental and ecological impacts, often leading to liquid and/or gas spills and losses (Shi et al., 2020). Environmental contamination, the use of lubricants in harvesting and food production machinery, and packaging can also cause food contamination from mineral oils and hydrocarbons (Grob, 2018). Additionally, enteric methane (CH_4_) is a short-lived climate pollutant with a warming effect 34 times greater than carbon dioxide.

The damage to the ozone layer and human health caused by the presence of gaseous hydrocarbons in the atmosphere, such as methane, benzene, and various polycyclic aromatic hydrocarbons, in addition to the contamination of soil and air due to hydrocarbon leaks from oil and gas plants and the emission of methane into the air and mineral oil hydrocarbons in the food industry, highlight the urgent need for the development of sensitive hydrocarbon detection techniques (Sampaolo et al., 2020; Wang et al., 2021; Martínez-Álvarez et al., 2022). Several methods have recently been demonstrated for hydrocarbon detection, including real-time polymerase chain reaction, genome sequencing-based techniques, hyperspectral remote sensing, reflectometric biosensing, optical spectroscopy, laser absorption spectroscopy (LAS), photoacoustic spectroscopy (PAS), and gas chromatography combined with various techniques such as mass spectrometry, vacuum ultraviolet detection, and flame ionization detection (Lawrence, 2006; Pejcic et al., 2007; Sgobba et al., 2020; Sampaolo et al., 2022). This Research Topic of Frontiers in Chemistry and Frontiers in Environmental Chemistry focuses on all aspects of research and development related to hydrocarbon detection techniques. The Research Topic includes seven original research works, which are summarized below:

In the first article, Zifarelli et al. report on an innovative sensor box for the simultaneous detection of methane and any targetable gas molecule M_x_ exhibiting absorption features in the infrared spectral range. The sensor operates on the principle of quartz-enhanced PAS and comprises two interconnected acoustic detection modules. The sensor box has been proven as extremely versatile for environmental monitoring as it simultaneously detects methane, nitric oxide (NO), and water vapor in indoor air, with limits of detection (LoD) of 48 parts per billion (ppb) and 11 ppb for CH_4_ and NO, respectively.

In the second study, Li et al. developed a field-deployable sensor based on tunable diode-LAS technology using a multi-pass gas cell for the simultaneous detection of carbon monoxide (CO) and nitrous oxide (N_2_O). The Particle Swarm Optimization-Kernel Extreme Learning Machine algorithm was demonstrated to better predict CO and N_2_O concentrations as compared to back-propagation neural networks and partial least squares regression (PLSR). LoDs of 0.25 ppb for CO and 0.27 ppb for N_2_O were obtained with averaging times of 24 and 38 s, respectively. Field deployment of the sensor for simultaneous detection of CO and N_2_O in the air was reported.

In the third original research article, Zhao et al. report on on-chip silicon-on-insulator waveguide CH_4_ sensors at 3.291 μm based on direct absorption spectroscopy (DAS) and wavelength modulation spectroscopy (WMS). By optimizing the waveguide cross-section structure, a high power confinement factor of 23% and a low loss of 0.71 dB/cm were demonstrated. LoD of 155 parts-per-million (ppm) by DAS and 78 ppm by WMS with averaging times of 0.2 s were obtained for a 2 cm-long waveguide sensor. Compared to chalcogenide waveguide CH_4_ sensors at the same wavelength, the reported sensor has the lowest waveguide loss and the lowest LoD.

In the fourth work in this Research Topic, Wang et al. employ a gas chromatography-mass spectrometry selective detector/flame ionization detector to detect 57 non-methane hydrocarbons (NMHCs) in the atmosphere at two coal chemical industrial parks in the Ningdong Energy and Chemical Industrial Base. This study identified industrial activities as the emission sources of alkanes, alkenes, acetylene, and aromatics. The propylene equivalent concentration method showed that alkenes predominated in the chemical reactivities of NMHCs. Alkenes and acetylene were the largest contributors to the potential for ozone formation. Ethylene, propylene, m/p-xylene, n-butane, 1-butene, propane, and acetylene were the major precursors in ground-level ozone formation.

In their study, Liu et al. developed a PAS sensor combined with a Herriott-type multi-pass cell to detect atmospheric CH_4_ traces. A DFB diode laser at 1,653 nm was used as the light source, and WMS was used to reduce the noise of the system. The sensitivity of the PAS was significantly improved by a factor of 13 in comparison with that of the single pass. A LoD of 116 ppb was obtained with averaging times of 84 s. The system was utilized for a 2-day test campaign to validate the feasibility and robustness of the sensor. The system provides a promising technique for online monitoring of greenhouse gases.

In the sixth contribution to this Research Topic, Bahr and Wolff present a PAS-based sensor for the detection of the methane isotopologues ^12^CH_4_ and ^13^CH_4_ at high concentrations. A 3,323 nm interband cascade laser was employed as the laser source, while the PLSR algorithm was selected for isotopologic data analysis of two-digit percentage level methane concentrations (25%–70%) in nitrogen. The results proved that PAS is a suitable method for the detection of ^12^CH_4_ and ^13^CH_4_ in highly concentrated methane, and therefore in undiluted natural gas samples.

Finally, in the seventh Original Research article, Mead et al. conducted field monitoring of methane and volatile organic compound emissions near an unconventional oil well development in Northern. A mid-infrared dual-comb spectrometer allowed quantification of methane, ethane, and propane in a single measurement with high time resolution and integrated path sampling. Using ethane and propane as tracer gases for methane from oil and gas activities, large emissions were observed during the drilling and milling phases, while emissions decreased to background levels during the flowback phase.
